# Effective treatment of steroid‐resistant immune checkpoint inhibitor pneumonitis with mycophenolate mofetil

**DOI:** 10.1002/rcr2.1356

**Published:** 2024-04-15

**Authors:** Nao Shioiri, Ryota Kikuchi, Itsuka Matsumoto, Kanako Furukawa, Kenichi Kobayashi, Shinji Abe

**Affiliations:** ^1^ Department of Respiratory Medicine Tokyo Medical University Hospital Tokyo Japan

**Keywords:** immune checkpoint inhibitor pneumonitis, immunosuppressants, lung cancer, mycophenolate mofetil, steroid

## Abstract

Insufficient evidence is available for treating steroid‐resistant immune checkpoint inhibitor pneumonitis (CIP). Although guidelines recommend the use of immunosuppressants, the efficacy of mycophenolate mofetil (MMF) has not been sufficiently verified. We report two cases of steroid‐resistant CIP treated with MMF. Both patients responded to initial treatment with prednisolone (PSL), but the CIP flared up repeatedly as the steroids were gradually tapered off. Upon receiving MMF in addition to PSL, their subjective symptoms improved, and the shadows gradually disappeared, allowing for a reduction in the steroid dose. Ultimately, no CIP recurrence was observed despite discontinuing PSL and MMF. Both cases were completely resolved by treatment with MMF. This indicates that MMF may be effective in treating steroid‐resistant CIP. In the future, the effects and safety of MMF should be investigated in large‐scale clinical trials targeting patients with steroid‐resistant CIP.

## Graphical abstract

Insufficient evidence is available for treating steroid‐resistant immune checkpoint inhibitor pneumonitis (CIP). Although guidelines recommend the use of immunosuppressants, the efficacy of mycophenolate mofetil (MMF) has not been sufficiently verified. Herein, we report two cases of steroid‐resistant CIP that improved with the co‐administration of mycophenolate mofetil (MMF).

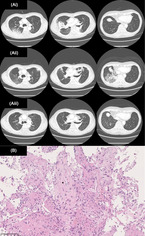

## INTRODUCTION

Immune checkpoint inhibitors (ICIs) are currently the standard treatment for lung cancer patients. ICIs may cause immune‐related adverse events (IrAEs) that are not observed with conventional chemotherapy or molecular targeted therapy. Immune checkpoint inhibitor pneumonitis (CIP) is one of the most common IrAEs, with a reported incidence of 5%–19% in lung cancer patients.^1^, ^2^ The first choice for CIP treatment is steroids, which are effective in most patients.^2^ However, some CIP cases are resistant to steroids. The mortality rate for patients with steroid‐resistant CIP is 34.6%–75.0%.^3^, ^4^, ^5^ Although guidelines recommend using immunosuppressants for steroid‐resistant CIP,^6^ evidence to support using immunosuppressants in these patients is insufficient. Furthermore, little evidence is available to support the effectiveness of mycophenolate mofetil (MMF) in steroid‐resistant CIP. Herein, we report two cases of steroid‐resistant CIP that improved with the co‐administration of MMF.

## CASE REPORT

### Case 1

A 72‐year‐old former smoker with cT2aN3M1c stage IVB lung squamous cell carcinoma was admitted to our hospital with dyspnea and cough. The patient did not exhibit extrapulmonary symptoms such as weight loss, arthralgias, muscle pain, or rash during the month before presentation. Although a complete response to pembrolizumab treatment was reached, the patient suffered from grade 2 CIP based on the Common Terminology Criteria for Adverse Events (CTCAE) version 4.0. Pembrolizumab treatment was discontinued due to pembrolizumab‐related pneumonitis, and the patient was receiving prednisolone (PSL, 50 mg/day) without any immunosuppressive drugs. The CIP was partially relieved by the steroid treatment. The steroid dose was reduced by 5–10 mg every 2 weeks and finally reduced to 5 mg/day.

**FIGURE 1:**
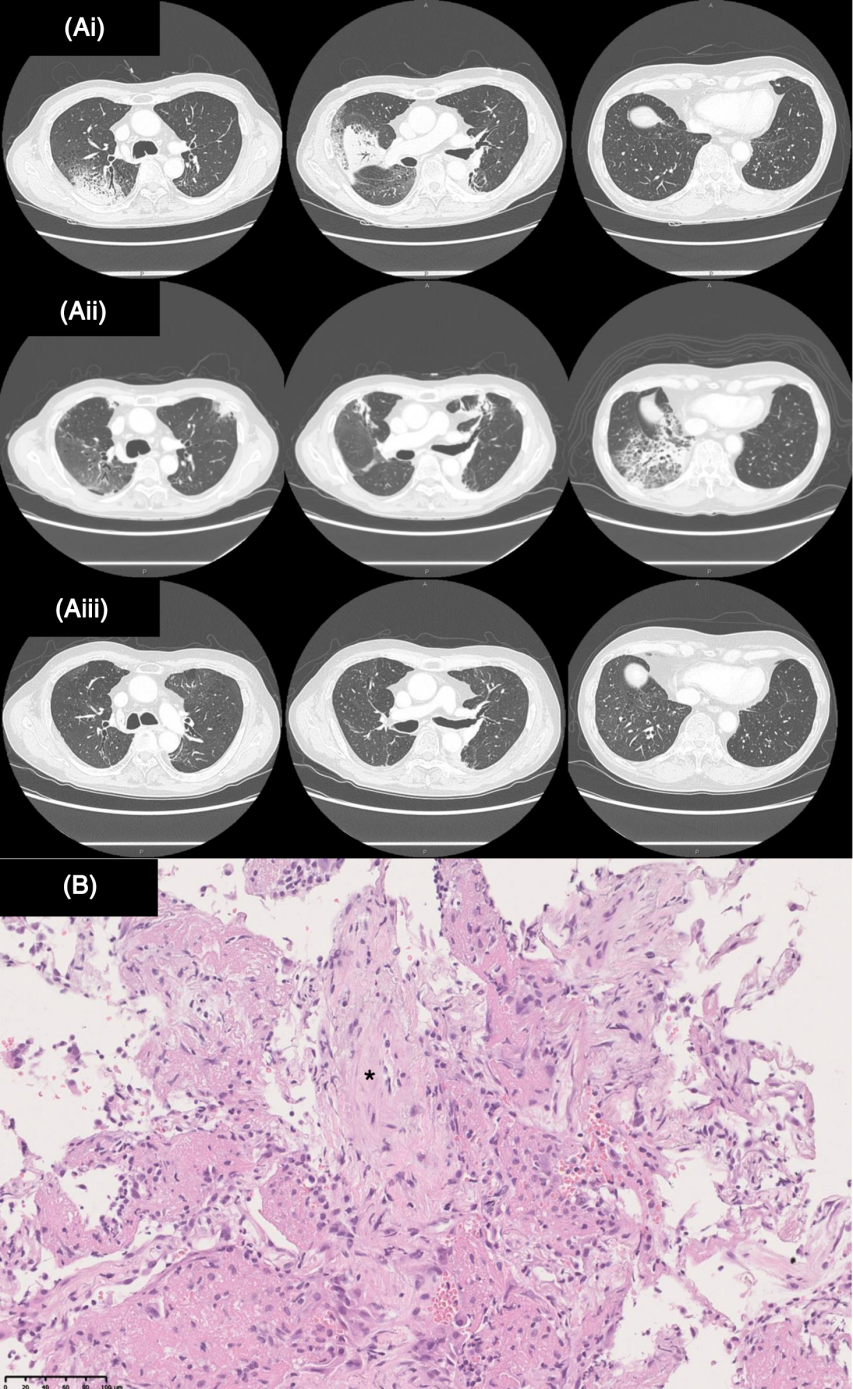
(A‐i) Chest CT 3 months before admission showing consolidation and ground‐glass opacities with an air bronchogram in the right upper and middle lobes. (A‐ii) Chest CT on admission showed decreased density of the infiltrative shadow in the right upper lobe but new infiltrative shadows in the right middle and lower lobe and left upper lobe. (A‐iii) Chest CT image obtained after 3 months of PSL and MMF treatment. The ground‐glass opacities and consolidations disappeared. B Microscopic findings showing lymphocytic infiltration into the thickened alveolar wall and organized intra‐alveolar lumen (asterisk) (haematoxylin and eosin staining). Scale bar = 100 μm.

On admission, the patient was hypoxemic (SpO_2_ 88% at room air) but had no fever. Blood tests revealed high white blood cell (WBC) (12,900/μL) and C‐reactive protein (CRP) (15.1 mg/dL) levels. Serum levels of lactate dehydrogenase (LD; reference range: <222 IU/L) and Krebs von den Lungen (KL)‐6 were normal. Serological tests for autoantibodies against specific antigens were all negative. Chest computed tomography (CT) revealed that a previous infiltrative shadow in the right upper lobe was partially attenuated, but both lung fields exhibited new bilateral diffuse infiltrative and ground‐glass opacification (Figure 1A–i, A‐ii). The bronchoalveolar lavage fluid was negative for microbes. Histological examination of a transbronchial lung biopsy specimen revealed organization and lymphocyte infiltration in the alveolar spaces (Figure 1B). No malignant findings were observed. Based on these findings, the patient was diagnosed with grade 3 ICI‐related steroid‐refractory organizing pneumonia caused by pembrolizumab.

CIP was previously difficult to control with steroids alone in this patient. Therefore, we proposed administering MMF. Only a few reports describe the efficacy and safety of MMF for CIP. Therefore, consent was obtained from the patient before MMF treatment. Furthermore, we received permission from our hospital evaluation committee to use MMF. Methylprednisolone (0.5 g/day) pulse therapy was initiated, followed by treatment with oral PSL (50 mg/day) and MMF (1000 mg/day). The steroid dose was gradually lowered over 12 weeks. MMF was administered for 12 weeks. The patient's clinical condition and chest radiographic findings improved without serious adverse events. Ultimately, both PSL and MMF were discontinued, and neither the CIP nor the lung cancer recurred (Figure 1A‐iii).

### Case 2

**FIGURE 2:**
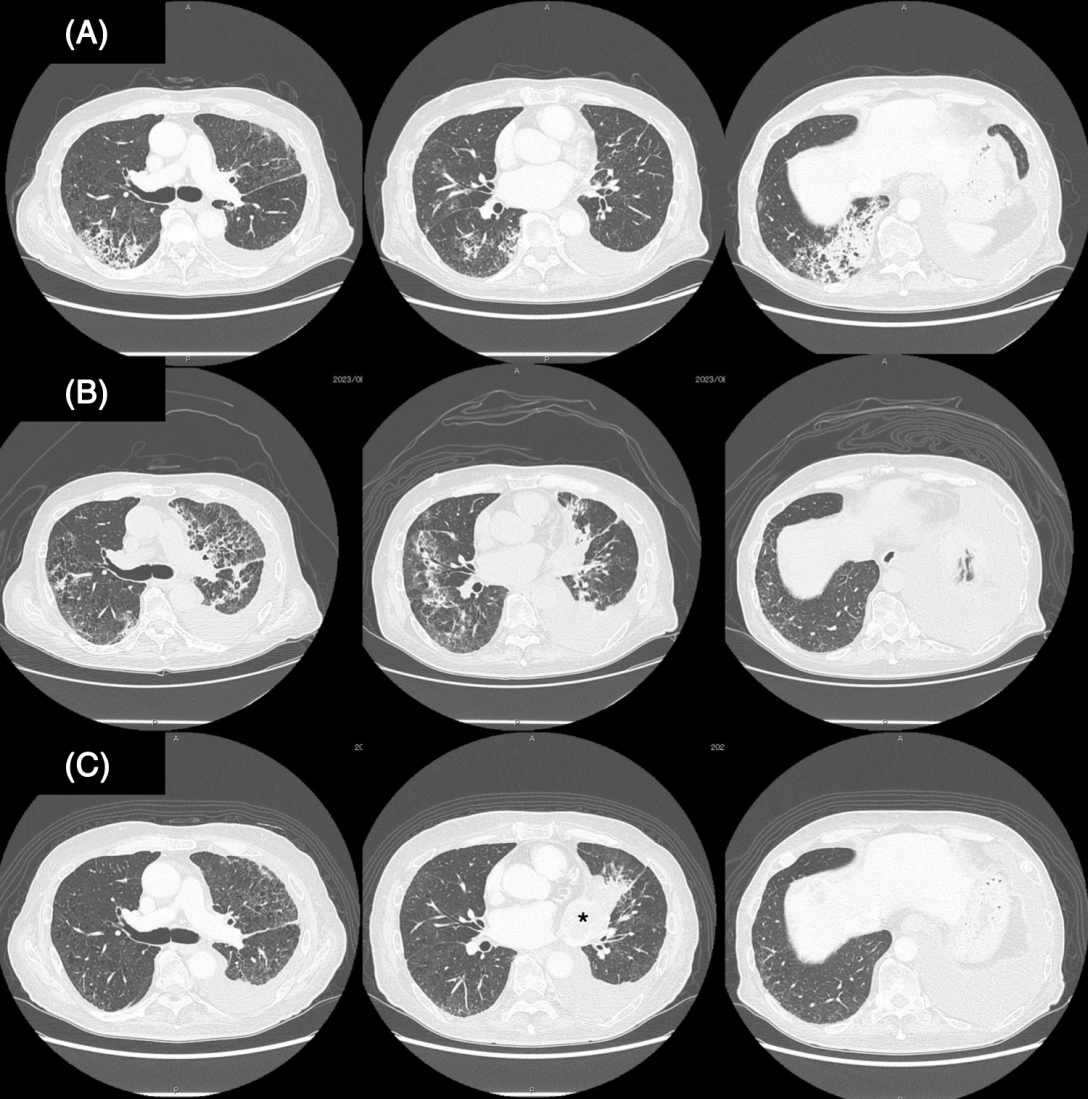
(A) Chest CT 3 months before admission revealing infiltrative and ground‐glass opacities in the right upper and lower lobes. (B) Chest CT on admission showed the decreased density of the infiltrative shadows in the right upper and lower lobes but new infiltrative shadows in the right middle lobe and the left upper and lower lobes. (C) Chest CT scan 3 months after PSL and MMF therapy showing the near disappearance of the consolidation and ground‐glass opacities. However, the mass shadow in the left hilar region expanded (asterisk).

A 73‐year‐old man was admitted to our hospital with dyspnea. Chest CT revealed a large left pleural effusion. The patient was diagnosed with cT4N2M1a stage IVA squamous cell carcinoma of the lung based on a systemic examination. The patient responded to pembrolizumab and achieved a partial response. After 23 cycles of treatment, the patient suffered from dyspnea. The patient's peripheral arterial oxygen saturation decreased (SpO2, 88% on room air). Laboratory tests revealed elevated WBCs (10,900/μL), serum CRP (7.6 mg/dL), and LD (231 IU/L) levels. Serum KL‐6 levels were normal. Serological tests for autoantibodies against specific antigens were negative. Chest CT revealed ground‐glass opacities and consolidation in the right upper and lower lobes (Figure 2A). No bacteria were detected in the sputum culture. The clinical course and imaging features showed that CTCAE grade 3 pembrolizumab‐related pneumonitis was suspected. Pembrolizumab was discontinued, and PSL (60 mg/day) was administered. PSL treatment partially alleviated the opacities, but CIP recurred when the dose was reduced to 10 mg/day (Figure 2B). CIP could not be controlled with steroids alone; thus, the patient was diagnosed with steroid‐resistant CIP. After obtaining informed consent and hospital permission for MMF treatment, PSL (60 mg/day) and MMF (1000 mg/day) were co‐administered. The patient's subjective symptoms improved and the shadows gradually disappeared. The prednisone dose was reduced by 10 mg every 2 weeks. MMF was administered for 13 weeks. No serious infections occurred during the treatment period. Although the lung cancer eventually expanded and malignant pleural effusion increased, the CIP improved. Furthermore, no CIP recurrence was observed, despite discontinuation of PSL and MMF (Figure 2C).

## DISCUSSION

We experienced two cases of steroid‐resistant CIP, which completely reversed after treatment with MMF in addition to PSL. Furthermore, we were able to discontinue the immunosuppressants with no CIP recurrence. Steroid‐resistant CIP is less effective with immunosuppressants than other IrAEs.^3^, ^4^, ^5^, ^7^ Infliximab has been used in most patients, but the response rate is only 0%–33%.^2^, ^3^, ^4^, ^8^ Therefore, the use of infliximab for steroid‐resistant CIP has recently been questioned due to its poor therapeutic efficacy.^5^ Nevertheless, the effects of immunosuppressants other than infliximab have not been sufficiently investigated. In particular, MMF treatment was only evaluated in a few patients. Jia et al. reported that administering MMF to patients with steroid‐resistant CIP did not improve the patients' conditions.^3^ However, another report demonstrated the effectiveness of MMF. The rate of sustained improvement in pneumonia was 83% (5 out of 6 patients) when MMF was used as the first immunomodulator for steroid‐resistant CIP.^4^ Furthermore, it has been reported that MMF is effective for treating steroid‐resistant IrAEs, including ICI‐associated liver injury that recurs during steroid tapering.^9^ Therefore, we decided to administer MMF, which is relatively effective. Based on our report, additional treatment with MMF may be useful for patients with steroid‐resistant CIP. Only retrospective data are available regarding the treatment of steroid‐resistant CIP. In the future, the effects of MMF should be evaluated in large‐scale prospective clinical trials.

MMF is also used for immunomodulatory treatment of interstitial lung diseases (ILD) other than CIP. Studies have shown that MMF is non‐inferior to oral cyclophosphamide therapy in reducing forced vital capacity decline in patients with systemic sclerosis‐associated ILD (SSc‐ILD).^10^ Moreover, in patients with fibrotic hypersensitivity pneumonitis, treatment with MMF was associated with improved diffusing capacity for carbon monoxide.^11^ The MMF doses used in these studies typically ranged from 1000 to 3000 mg/day. Furthermore, in a survey using Japanese hospital billing data, the most common initial and maximum dose of MMF for patients with SSc‐ILD was 1000 mg/day.^12^ Based on these results, we adjusted the MMF dose to 1000 mg/day for the treatment of CIP. However, determining the appropriate usage and dosage of MMF for CIP will require further study.

ICIs exert antitumor effects by activating immune cells. Thus, IrAEs may be due to an excessive immune response.^13^ MMF suppresses DNA synthesis by inhibiting inosine monophosphate dehydrogenase in the de novo synthesis of intracellular nucleic acids. Consequently, it selectively suppresses the proliferation of lymphocytes.^14^ Although the detailed mechanism is unknown, MMF may suppress the proliferation of self‐antigen‐specific T lymphocytes, which underlies the onset of CIP to cause an immunosuppressive effect.

In Case 2, CIP improved, but the lung cancer progressed. This may be due to excessive immunosuppression. In the management of IrAEs, immunosuppression may negatively impact the underlying cancer.^15^ Therefore, it is necessary to investigate whether the use of immunosuppressants, including MMF, prolongs cancer prognosis in patients with steroid‐resistant CIP.

## AUTHOR CONTRIBUTIONS

N. S. and R. K. wrote the manuscript. All authors contributed to editing of the manuscript and approved the final version of the manuscript.

## CONFLICT OF INTEREST STATEMENT

None declared.

## ETHICS STATEMENT

The authors declare that appropriate written informed consent was obtained for the publication of this manuscript and accompanying images.

## Data Availability

The data that support the findings of this study are available on request from the corresponding author. The data are not publicly available due to privacy or ethical restrictions.
